# What Type of Body Shape Moves Children? An Experimental Exploration of the Impact of Narrative Cartoon Character Body Shape on Children’s Narrative Engagement, Wishful Identification, and Exercise Motivation

**DOI:** 10.3389/fpsyg.2021.653626

**Published:** 2021-07-12

**Authors:** Dar Alon, Caio Victor Sousa, Amy Shirong Lu

**Affiliations:** Health Technology Lab, College of Arts, Media and Design, Bouvé College of Health Sciences, Northeastern University, Boston, MA, United States

**Keywords:** body shape, character, narrative, story, children, active video game, physical activity, animation

## Abstract

**Background:**

The incorporation of narratives helps to enhance children’s engagement in active video games (AVGs), thus increasing moderate-to-vigorous physical activity (MVPA). Specific narrative elements, such as the visual representation of the characters’ body shape, have been rarely manipulated to explore their role in modulating children’s narrative engagement (NE) and exercise motivation.

**Objective:**

To investigate the effects of character body shape manipulation (overweight/obese, average, or athletic slim) on children’s narrative immersion (NI), NE, wishful identification (WI), as well as their mediating effect on AVG and PA motivation.

**Methods:**

Children ages 8–12 years old (*N* = 87) were randomly assigned to watch a 15-min animated video (designed for an existing AVG) in which the main characters had an overweight/obese, or average^∗^, or athletic slim body shape (all other elements were identical). Children’s NI, NE, WI, and AVG and physical activity (PA) motivation were then assessed.

**Results:**

Controlling for social desirability, the analysis indicated that participants with a BMI of greater than the 75th percentile had a significantly higher NI, NE, WI, and PA motivation when video characters were set to the overweight/obese condition, than they did for video characters set to the average or athletic slim conditions. On the other hand, children of equal or less than the 75th percentile exposed to the average character body condition had a greater NE, WI, and PA motivation than overweight/obese or athletic slim conditions. A mediation analysis with structural equation modeling indicated that NE mediated the effect between character body shape and AVG and PA motivation.

**Conclusion:**

Narrative cartoon characters that mirror the target participant’s body shape can increase NE, which in turn mediates AVG and PA motivation. Content producers should identify optimal strategies in character body shape design to encourage children of different weight status to participate in PA with engaging stories to maximize health narratives’ persuasive potentials.

^∗^The term average in this sense is not in reference to the national average body weight, but rather an average of the body weights represented in conditions A and C.

## Introduction

Entertainment media narratives have great potential in shaping children’s perceptions and behavior (Strasburger et al., 2013). Characters are a primary component of persuasive media narratives (Lu et al., 2012a). Thus, it is crucial to effectively present these characters in order to maximize the persuasive goals and induce behavioral change in children (Lu et al., 2012a). With the current childhood obesity epidemic (Wang et al., 2020), it is even more important to examine how the body shapes of media characters should be presented to children when the goal is to encourage them to maintain or lose weight.

In this context, the “ideal body shape” of media characters portrayed in media is conveyed to children across different developmental stages and may be a motivational factor in altering their own body shapes (Harrison, 2000b; Field et al., 2005). On the other hand, these portrayals may also lead to the stigmatization of obesity (Latner et al., 2007). Therefore, it is critical to examine various types of mediated representation of bodies and their potential influence on children. Most mediated body studies have focused on existing characters in mass media narratives (Baker and Raney, 2007; Northup and Liebler, 2010; Tzoutzou et al., 2020). Some altered the character weight featured in transient media clips such as an advertisement to study its effect on children’s perception of the health quality of the product (Castonguay et al., 2019). Few studies have experimentally manipulated the visual representation of the characters’ body shape or weight status in an existing narrative, or a story, which permeates media content.

As a crucial element in narrative persuasion, characters, especially their body shapes, have an important role to motivate or deter children to exercise. For children with overweight or obesity, the goal of a persuasive narrative would be for their weight loss, resulting in a body shape of healthy body weight. How should the characters’ body shape look in the narratives for them? Research so far has not provided a consistent answer to this question. The tailoring theories suggest that creating characters that look similar to the actual self, i.e., overweight/obese for overweight/obese children, may improve motivation to increase PA for better health outcome (Kreuter et al., 2013). On the other hand, the potential effects of observing or acting as overweight/obese characters may have adverse effects on the players. In addition, self-perception theory suggests that people rely on external cues (e.g., a game character) to infer their internal states (e.g., attitudes and emotions) and behave in accordance with the inference (Bem, 1972). From a Proteus effect perspective (Yee and Bailenson, 2007), watching an overweight or obese character in a narrative may cause the audience to be less likely to exercise while a character of ideal weight might do the opposite. For example, a recent study has suggested that showing children athletic slim and toned characters, or an ideal self-image, would motivate them to have better active video game (AVG) performance (Li et al., 2014).

Thus, the question remains: What type of body shape moves children? This study aims to address this question through an experimental study of character body shape manipulation in an animated narrative series among 8–12-year-olds.

## Literature Review

This section will review a series of interdisciplinary literature. We will first situate this study in the larger public health context of the importance of reducing child obesity in terms of both adverse physical and psychological effects, a goal that this study shares. Then we will discuss the disappointing results of engaging children in physical activity (PA) and the potential benefits of AVG in increasing PA. In the next section, we will discuss how children do not play with AVGs for a long enough duration to have an impact and the potential positive impact of narrative that captures the child’s attention with interesting plots and extraordinary characters in PA promotion via AVGs as well as reviewing several potential mediators of the narrative effect such as narrative immersion (NI), narrative engagement (NE), and wishful identification (WI). We also explore gender differences in the psychological effects of being overweight and obese. Children may have emotional responses to the body images of the cartoons that lead to dissatisfaction and potentially a decrease in motivation to participate in PA. Last but not least, we will review the visual representation of mediated character’s body shapes and the effect of children’s exposure to these body shapes.

### Child Obesity, Physical Activity, and Active Video Games (AVGs)

In the United States, approximately 16.6% of children are overweight, and an additional 18.5% of children are considered obese (Fryar et al., 2018; National Center for Chronic Disease Prevention and Health Promotion, 2019). Childhood obesity (CO) has many negative influences on the developmental trajectory. It continues into adulthood (Whitaker et al., 1997), shortens lifespan (Danaei et al., 2009), increases multiple cancer risks (National Cancer Institute, 2017), impedes functional ability (Li et al., 2008), and diminishes quality of life (Swallen et al., 2005). Children with overweight or obesity are more likely to experience various psychosocial problems, such as aggressive and disruptive behavior (Puder and Munsch, 2010), and are more likely to be victims of bullying and teasing (Schwimmer et al., 2003).

Physical activity (PA) helps to prevent childhood obesity and has significant psychological benefits on children with obesity, including improvement in emotional well-being, self-perception, and self-confidence (Estabrooks et al., 2008; Katzmarzyk et al., 2015; Romero-Pérez et al., 2020). United States PA guidelines recommend 60+ min of age-appropriate, enjoyable, mostly moderate, or vigorous daily PA (MVPA) for children (U.S. Department of Health and Human Services, 2018). Yet, few children meet the guidelines (National Physical Activity Plan, 2014). The majority of PA interventions (e.g., home, school, or community-based), have yet to achieve sufficient engagement from children (Metcalf et al., 2012; Thivel et al., 2018). The study seeks to identify child-friendly PA interventions that are both engaging and sustainable.

An alternative PA intervention to promote exercise comes in the form of exercising using AVGs, also known as exergames, which are video games that require movements that mimic “real-life” exercise (Bailey and McInnis, 2011). Unlike traditional PA, AVGs gamify the component of exercise via various gaming consoles (e.g., *Wii Sports Resort*, *Xbox Kinect Adventures*). Children are more likely to associate AVG and exercise with fun and engaging gaming experiences. AVGs have been demonstrated to motivate children to engage in moderate-to-vigorous PA (Hwang et al., 2019; Williams and Ayres, 2020). Previous systematic reviews have found that AVG use significantly increases duration of vigorous PA among children (Lu et al., 2013; Gao and Chen, 2014) and AVGs have a small to medium effect of on BMI reduction (van‘t Riet et al., 2016).

### Narratives for PA Promotion via AVGs

Despite their seemingly engaging qualities, AVGs are less likely to be played for a long periods of time (Graves et al., 2010; Lyons et al., 2011). A potential reason for this reduced long-term engagement is that AVGs seldom incorporate narratives (Lu and Kharrazi, 2018) and are then perceived to be less enjoyable than sedentary games.

The use of narrative could be a solution to this problem. Narratives, defined as two more events occurring in succession (Rimmon-Kenan, 2003), have been identified as an integral component of the player experience and motivation (Bateman, 2006; Yee, 2016). The addition of a narrative to existing AVGs may increase user motivation and ultimately lead to increased PA (Lu, 2015; Sousa et al., 2020).

Not all narratives elicit the same motivational effects in children. Previous studies found that narratives with interesting plots, including cliffhangers, and extraordinary characters are capable of promoting PA engagement in children aged 8–12 years (Lu et al., 2016b). In another study, children preferred the narrative cartoon as opposed to non-narrative cartoon and reported enjoyment of the story and exercise (Davis et al., 2016). Children in the 8–12-year age group, across all weight, race, and gender groups, also preferred a fantasy genre of dystopian science fiction (Lu et al., 2019). Indeed, the most popular reading topic among boys and girls is the science fiction genre (Sturm, 2003).

Most of the previous studies on obesity combating narratives have targeted the 8–12-year age group. Without intervention, children with obesity in this age group are highly likely to become obese young adults (Whitaker et al., 1997). Therefore, examining outcomes in a high-risk population is especially important (Foster et al., 2010). Interventions have had effects primarily among the people with overweight and obesity (McMurray et al., 2000). Implementing narrative AVGs in higher-risk groups should result in greater positive outcomes (Ledoux et al., 2010). Children younger than eight have cognitive limitations in responding to survey questionnaires or interview questions (Borgers et al., 2000). Children older than 12 have entered early adolescence and will be subject to many physical, mental, emotional, and social changes that may require different intervention strategies (Centers for Disease Control and Prevention (CDC), 2010).

### Narratives Immersion, Narrative Engagement, and Wishful Identification: Mediation Effects

Researchers have found that narratives Immersion, NE, and WI are potential mediators that improve or deteriorate the narrative effect. NI was adapted from the term “narrative transportation” (Green and Brock, 2000), which originally was defined as the story’s ability to “transport” audiences to a different world. Due to the highly interactive and immersive qualities of videogames, the term “NI” was coined to describe the involving nature of videogames among children. We have also found that children had a better understanding of the term “NI” than “narrative transportation” in our interaction with them over the research sessions. The NI has been found to underlie the stories’ motivating effect in PA (Sousa et al., 2020).

Narrative engagement, a relevant concept to narrative transportation/immersion, is based on the notion that NE is a process of constructing mental models of narrative events (Busselle and Bilandzic, 2009). While NE tends to be highly correlated with narrative transportation/immersion, it includes four additional dimensions of NE: narrative understanding, attentional focus, emotional engagement, and narrative presence (Busselle and Bilandzic, 2009). Both narrative immersion and engagement may be associated with narrative health persuasion outcomes, e.g., increasing children’s motivation to exercise. Therefore, narrative immersion and engagement may mediate the effect of the character body shape manipulation on children’s exercise motivation.

Wishful identification, defined as the desire to become or mimic actions of characters in the narrative (Hoffner, 1996), may influence exercise motivation as well when the narrative character’s body shape has been changed. Due to the desirability of athletic slim characters, a child may have a higher WI with athletic slim characters, who represent societal ideals. This corresponds to the self-perception theory (Bem, 1972) that argues that children may rely on the character’s body shape to infer their internal states and behave according to the inference. Seeing athletic slim characters may therefore encourage children to behave as them by exercising regularly. On the other hand, according to tailoring literature, children with overweight or obesity are more likely to identify with characters that look like themselves and thus would perceive these characters and the stories to be more relevant to themselves as well as have higher WI with them (e.g., want to act like the overweight or obese characters). A study of AVG has found that customized avatars with idealized body shapes (as opposed to realistic features) decreased the players’ AVG performance (Koulouris et al., 2021).

In other words, seeing characters similar in body shape for children with overweight/obesity may enhance the perceived self-relevance, enabling them to become more immersed in a narrative, resulting in a higher WI with the characters. On the other hand, seeing characters engaging in PA in a body shape that the children wish to resemble, may also enable them to be more engaged with the narrative and having a higher WI. Either way, the increased engagement with narratives and WI has the potential to motivate them to participate in higher PA to combat obesity. More research is needed to determine whether similar or aspirational body shapers are more likely to encourage children to engage in PA.

### The Visual Representation of Media Characters’ Body Shapes and the Effects of Exposure to Them

The visual components of the video narratives added to AVGs, such as the body shape of characters, may affect AVG motivation and amount of time spent engaging in PA. Narratives, especially those with vivid imagery, may affect players’ attention and adherence to health messages, such as encouraging PA (Baranowski et al., 2013), even more so than textual cues. However, this effect is dependent on the valence of emotional response users have to these images (Houts et al., 2006). One notable visual cue in narratives is the characters’ body shape, which can potentially modify children’s health-related outcomes (Common Sense Media, 2015).

The body shape of characters may also decrease motivation to engage in PA as well. The body shape is an inextricable aspect of contemporary American media, partially due to the prominence of character-based stories, and can contribute to negative psychological effects (Puhl et al., 2013), which can lead decreased time spent exercising (Han et al., 2018). The portrayal of overweight characters in American media often perpetuates stigmatization of obesity, thus negatively affecting overweight/obese children (Puhl et al., 2013). In adolescent targeted media, such as cartoons, movies, and books, children deemed overweight characters to be unattractive, less loving, less physically healthy, and less intelligent; children tended to classify such characters as “bad” (Ata and Thompson, 2010). Thus, the explicit body shape of the character depiction may provide implicit messages and associations concerning the quality of the character to children.

The body shape, media representation, and subsequent associations of the characters in media differs by gender as well. In cartoons, female characters were four times more likely to be depicted as underweight; overweight female characters were more likely to be presented as unintelligent or unhappy (Latner et al., 2007). For men, over the past 30 years, action figures have grown substantially in muscle size, correlating with decreased body esteem and an increase in depression in male audiences (Barlett et al., 2008; Martins and Harrison, 2012). Being exposed to characters that are predominantly athletic slim (for women) or muscular (for men) instead of characters with a variety of body shapes can lead to unsatisfactory body self-perception and a lack of self-esteem in both boys and girls (Barlett et al., 2008). For both genders, positive traits, such as being referred to as “good looking” were addressed toward slim characters (Tzoutzou et al., 2020).

The portrayals of the ideal body shape in both genders could lead to internalization, which causes body dissatisfaction and even lead to a decrease in motivation to participate in PA. A previous study demonstrated that people are especially susceptible to internalizing media messages about the idealized body shape, as determined by an internalization score. Those who were presented with a high internalization score were more likely to report higher body dissatisfaction (Dye, 2016). The body dissatisfaction, reinforced by ideals presented in media, can further manifest in a lower health-related quality of life, including physical ability and a decrease in motivation to participate in PA. Such detrimental effects may extend to adulthood. For example, in a self-reported adult study, exercise avoidance motivation was influenced by weight stigma and contributed to the negative correlations between body weights and PA (Han et al., 2018). Furthermore, a positive discrepancy between reported and actual self-figure was inversely related to health-related quality of life (Petracci and Cavrini, 2013).

For younger girls specifically, the internalization of the appearance ideals conveyed through characters, as represented in conversations with friends and criticism by peers, was significantly related to body dissatisfaction (Jones et al., 2004), which, as previously mentioned, can result in decreased PA motivation. Boys also suffer body dissatisfaction based on comparisons with friends, comments by peers, and their own body shape (de Vries et al., 2019). Furthermore, children’s media also perpetuate internalization of obesity stigmatization and the media use for both genders has been found to be correlated with a more negative attitude toward girls and boys with overweight and obesity and demonstrated the negative internalizing role an idealized depiction of both genders plays (Latner et al., 2007).

The negative effects of internalization are further highlighted through the narrative persuasion theory, which states that narratives can have an impact on beliefs, behaviors, and attitudes (Green and Brock, 2002). Accordingly, a narrative promoting certain body images or ideals may also shape the behaviors and beliefs of the audience. For both boys and girls, the amount of time engaging with idealistic images predicted low self-esteem and participation in diets to lose weight (Harrison, 2000a). Although this exposure to idealized images can motivate both genders to improve their body shapes, along with the effects of low self-esteem and body desirability produced by such exposure, there may also be undesirable aspects to body shape improvement, including unhealthy mechanisms for weight loss (Harrison, 2000a). In addition, as postulated by the social cognitive theory, learning occurs in a social context with a dynamic and reciprocal interaction of the person, environment, and behavior (Bandura, 1986), observational learning can occur from a model (Schunk and DiBenedetto, 2020) from a narrative. If these narratives provide a model that perpetuates unattainable, yet desirable body shapes, and unhealthy mechanisms to achieve said body, audiences who emulate this model may adopt these unhealthy behaviors. The negative effect on self-image and perception is also physically damaging as it can result in reduced motivation to engage in PA, ultimately demonstrating that body shape of various media figures has a key impact on self-perception (Petracci and Cavrini, 2013; Rideout, 2015).

Therefore, building upon previously research, we plan to create a narrative in which the visual components, specifically the body shape of the characters, resemble the viewer with a goal to increase PA. More specifically, to explore the influence of the variation of narrative character’s body shape on 8–12-year-old children’s exercise motivation (AVG and PA motivation), we manipulated the character’s body shape and examined their NI (Lu et al., 2012b), NE (Busselle and Bilandzic, 2009), and WI (Hoffner, 1996) that may be affected by manipulation of character body shape and explored how these factors may influence children’s motivation to exercise as a result of watching this animated narrative.

Due to the fact that WI is based on the notion that the character’s body shape matches that of the children, we divided the participants into two groups: children with a BMI greater than the 75th percentile vs. a BMI equal or less than the 75th percentile. Therefore, we could compare the effects of the different character body conditions on children of different weigh classes. The 75th percentile BMI marker is also correlated with onset of certain conditions, such as asthma and heightened allostatic load (Davis et al., 2007), which added further clinical relevance to this division.

We hypothesized that presenting the children with characters with similar character body shapes would ultimately enhance NI, NE, and WI, all of which in turn will increase PA and AVG play motivation.

## Materials and Methods

### Narrative Development

To investigate the effect of character body shape on the aforementioned outcomes, we presented children aged 8–12 with a single animated narrative video in which we had varied the body shapes of the main characters to three conditions (overweight/obese, average, and athletic slim). These three conditions were chosen to represent a graded scale of the body shape spectrum presented in media narratives, from the idealized body shape to characters that more closely resemble the overweight/obese population our research team works with. As part of a pilot study, children were presented with sample images of potential characters representing each condition and asked for their opinion. From their answers, we noted that the expression “thin” should be avoided and therefore chose “athletic slim” to avoid a negative connotation for children of the body shape. Furthermore, a team of research assistants were also interviewed about the perception of body shapes of three different conditions and contributed to the current naming convention.

A professional media production company, FableVision, was hired to create a 15-min animation about a dystopian Sci-Fi narrative, *Ataraxia*, that incorporated AVGs in the plotline. In *Ataraxia*, the plot concerns a dystopian future in which a twin brother and sister, who can absorb and take away people’s pain, are kidnaped by an evil dictator to create an army of indestructible soldiers. During the *Ataraxia* narrative, the player character, who grows up with the twins, begins to develop superpowers through exergaming and must train to hone their powers to aid in saving the twins and saving Ataraxia from various villains. The *Ataraxia* series was later developed into a six-season 72-episode animated series.

**FIGURE 1:**
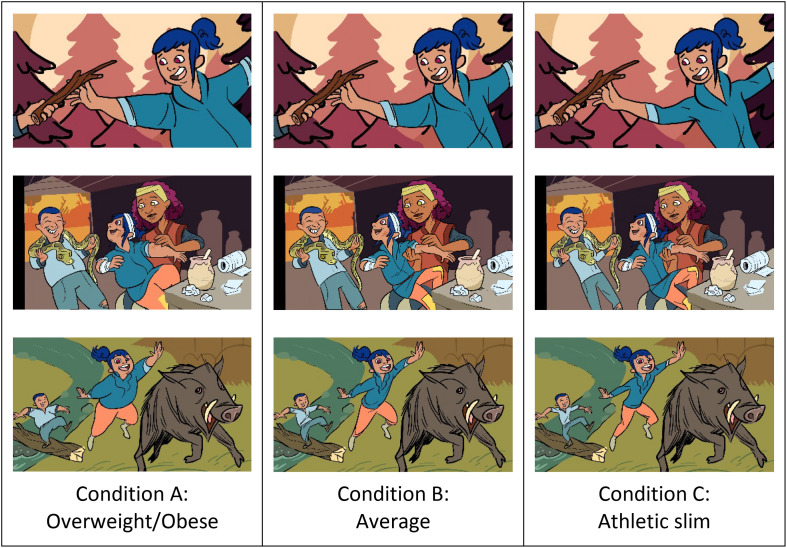
Image stills taken from the narrative trailer for each of the three body shape conditions. Produced by FableVision Studios for Northeastern University. Reproduced with permission from Northeastern University.

During the animation production, the production team created multiple layers of different animation panels to allow various modifications of different elements. This allowed for varying each element of a character (i.e., body shape independent of any other alterations such as hair color, background settings). With this approach, three different character types based solely on body shape (all other elements were kept equal) were created by a single animator to ensure a consistent visual style: overweight/obese (condition A), average (condition B), and athletic slim (condition C; see Figure 1). The audio was also kept identical across the conditions.

### Recruitment

We partnered with a charter school and eight different youth centers in the Greater Boston Area. Site coordinators helped to distribute information sheets to the parents of 8–12-year-old children attending afterschool activities. Research assistants (RAs) also placed posters/flyers with a brief overview of the study on stationary bulletin boards in the school and at the centers.

Regardless of the recruitment method, interested parents completed a screening questionnaire either online or in person, including parent contact information and basic demographic and physical information regarding their child. If they completed the questionnaire in person, it was then returned to their afterschool coordinators, who later passed them to the research team. All parents and children also completed consent and assent forms to participate in this project.

All data collection took place between January 8, 2019 and June 17, 2019. Inclusion criteria included the following: children who were between 8 and 12 years, able to speak and understand English, and able to complete the protocol. Exclusion criteria included the following: children who did not speak English, who had an intellectual disability (preventing them from understanding the narrative video), or who had a physical disability (preventing them from participating in AVGs).

After RAs obtained completed consent and assent forms from the site coordinators, they visited the sites to conduct further assessments of the children. The children’s height and weight were measured by the RAs two times: height (to the nearest 0.1 cm) using a ShorrBoard (Weight and Measure, LLC, Olney, MD, United States) and weight (to within 0.1 kg) using a SECA scale (SECA Inc., Chino, CA, United States). A third measurement was taken if there was more than a 0.2 cm (height) or a 0.2 kg (weight) difference between the first two measurements. Afterward, the demographic information previously provided was confirmed. Body mass index was calculated using the mean of height and weight measurements (BMI; kg/m^2^) and using the CDC growth charts (Kuczmarski et al., 2000). The children were then randomly assigned to watch one of three animation clips: one-third assigned to condition A, one-third to condition B, and one-third to condition C. The height and weight assessment, randomization, and actual data collection were within 1–2 weeks to ensure the timeliness and accuracy of the measurements.

### Procedure

To ensure children provided us with their unfiltered opinions, we used as the primary data collection site the media classrooms of their afterschool facility, where they usually gather to watch TV or play video games. Before watching the video, children were directed away from their afterschool activities and invited into the room to sit in chairs pre-arranged in front of a high-definition TV set. They were then told that the study was about their opinions of an animation movie created to encourage them to exercise more. They were told that the RAs would ask them for their opinions in a questionnaire that they would complete and that there was no right or wrong answer to the questions. The RAs told them that they should feel free to express themselves confidentially in the questionnaires. They were also reminded that they could stop their participation at any time. Children were then placed in groups of 4–15, depending on the room size, time of the visit, the afterschool program’s schedule, and the child participant’s availability.

### Questionnaires and Scales

After watching the animation clip, children were handed hard copies of questionnaires consisting of the following: a demographic questionnaire (age, sex, race, and ethnic background) and a psychological survey questionnaire measuring NI (Lu et al., 2012b), NE (Busselle and Bilandzic, 2009), WI (Hoffner, 1996), AVG motivation (a five-item scale developed for this project), PA motivation (Kendzierski and DeCarlo, 1991), and social desirability (SD) (Reynolds and Paget, 1983).

The NI scale (Lu et al., 2012b) was adapted from the original 11-item transportation scale (Green and Brock, 2000). One item (“I had a vivid image of [character name]”) was removed because the original scale was about transportation via textual media. Thus the original narrative transportation scale has been adapted into a “NI” scale, which captures all of the essential information of the original one to measure children’s response to visual narrative media (Lu et al., 2012b). The remaining 10-items had then been repeatedly implemented with children in a series of studies among children (Lu et al., 2012b, 2016a; Sousa et al., 2020). Through the children’s feedback during the study sessions, the RAs documented their questions about the questions and explained to them the meaning of the questions to ensure the scale to be child friendly for a better understanding. As a result, this effort resulted in the original scale language being modified slightly to the NI scale to ensure children’s understanding while retaining the original meaning.

Given children’s limited understanding of the Likert scales and the typical field practice in psychology (Mellor and Moore, 2013), we have also reduced the choice options from 7-point to 5-point, with 1 = disagree, 3 = neutral, and 5 = agree. Sample questions for NI include “I was completely involved in the story while watching it” and “I wanted to find out how the story ended” (Cronbach’s α = 0.544 initially and later = 0.610 after removal of two additional reversed-coded items, elaborated later).

Similarly, the 12-item NE scale was also adapted from the original study with adults (Busselle and Bilandzic, 2009) to be used for children. Minor changes were implemented to ensure that the questions were understood. Sample questions for NE include “I understood the story” and “I understood the characters,” with 1 = disagree, 3 = neutral, and 5 = agree (Cronbach’s α = 0.695).

The 3-item wishful identification (WI) scale was adapted without any modification as it was originally developed for children (Hoffner, 1996) with a 5-point scale. Sample questions for WI included “I would like to do the kinds of things he/she does in the story” and “He/she is the sort of person I want to be like,” with 1 = disagree, 3 = neutral, and 5 = agree (Cronbach’s α = 0.750).

The assessment of AVG play motivation (AVGM) is based on a 5-item scale developed for this project. The scale assessed the degree of children’s willingness to play an AVG if the story is included as its plot. Sample questions include “I intended to play this AVG” and “I plan to exercise through this AVG,” with 1 = disagree, 3 = neutral, and 5 = agree (Cronbach’s α = 0.792).

The PA motivation (PAM) employed a 16-item scale adapted from the validated exercise enjoyment scale (Kendzierski and DeCarlo, 1991), Sample questions include “(1=) (PA) is no fun at all” versus (5=) (PA) is a lot of fun;” and “(1=) (PA) is not at all exciting (=1)” vs. “(5=) (PA) is very exciting” (Cronbach’s α = 0.883).

The 9-item SD scale (Reynolds and Paget, 1983) was used to measure the importance of social approval in children. This variable was included because we wanted to control for demand characteristics and ensure that the children were not answering the questionnaires in a way to appease the researchers. Statements such as “I am always kind” and “I tell the truth every single time” were presented in an agree/disagree gradient scale, with 1 = disagree, 3 = neutral, and 5 = agree (Cronbach’s α = 0.841).

As children completed the paper copies of questionnaires, RAs observed their attention to the questionnaire completion and answered questions from them. They also took notes of children who simply answered all questions without reading them (e.g., selecting “1” or “5” for all questions on multiple pages or completing the questions in extremely short period of time (e.g., finishing over 50 questions in less than 3 min), which suggested the lack of validity for these answers. After children have completed the questionnaires, children were given a $25 gift card for their participation, thanked, and brought back to their afterschool activities. The RAs then collected the questionnaires and entered them into the database. All data entries were triple checked to ensure accuracy. The questionnaires from those less attentive participants were identified and later removed for analysis.

## Results

### Statistical Analysis

To compare the perceptions of conditions A, B, and C by children of different weight status, we dichotomized our participant population using the 75th percentile of BMI, which also happened to have resulted in a median split of the total sample as a cut-off point. The Shapiro–Wilk and Levene’s tests were applied for normality and homogeneity, respectively. All variables presented a normal and/or homogeneous distribution and were analyzed using parametric tests. Characteristics of the sample were compared between the group ≤ 75 and >75 BMI percentile with an independent *t*-test. The Cronbach’s Alphas were calculated for all of the scales (reported in section “Materials and Methods”).

Since the Cronbach’s Alpha of NI was relatively low (0.544), we then conducted a factor analysis for NI and after excluding two items (“When I was watching the story, other activity going on in the room around me was on my mind.” and “After the story ended, I found it easy put it out of my mind.”), the new Cronbach’s Alpha was 0.610. Nevertheless, due to the high correlation between NI and NE (*r* = 0.716, *p* < 0.001), which was routinely observed over our child sample (Lu et al., 2012b, 2016a; Sousa et al., 2020), we decided to examine both variables as outcomes of narrative persuasion, but only include NE in the mediation analysis. The scales were thus all averaged for additional analysis.

General linear models with two factors (two-way ANOVA) were ran for each outcome. BMI percentile (≤75th/>75th) was added as fixed factor, and Condition as a random factor (conditions A, B, and C, were coded as 1, 2, and 3, respectively). When interactions were found (*p* < 0.05), pairwise comparisons were applied to further identify the differences. The hypothesis of sphericity was verified by Mauchly test, and when violated, the degrees of freedom were corrected by the Greenhouse–Geisser estimates.

Structural equation modeling (SEM) was conducted using Mplus 7 (Muthén and Muthén, 2018) with maximum likelihood estimation. Given the relatively small sample size in relation to the number of individual variables (Bollen, 1989), we did not adopt a latent variable model. Instead, a path model using manifest variables was developed to examine the mediating role of NE and WI of the effect of character body shape manipulation (IV) and children’s BMI percentile (IV) on children’s AVG (DV) and PA motivation (DV). We used Hu and Bentler’s (1999) criteria for model fit indices. More specifically, to indicate a good model fit, both Comparative Fit Index (CFI) and Tucker Lewis index (TLI) should be 0.96 or higher; the root mean square error of approximation (RMSEA) should be 0.06 or lower; the standardized root mean square residual (SRMR) should be 0.08 or lower. All path coefficients reported later were standardized estimates, which were indicative of effect sizes (Muthén and Muthén, 2018).

The statistical significance level was set at 5% (*p* < 0.05). The independent *t*-test, and ANOVA models showed a power of analysis of 79% or higher considering a moderate effect size and the final sample size (*n* = 87). All non-SEM statistical procedures were carried out using the Statistical Package for the Social Sciences (SPSS) 26 (IBM Corp, 2020) and GraphPad Prism 8.4.2 (GraphPad Software, 2020).

### Demographic Information

Data was originally collected from 96 children. Of them, nine children were identified as improperly answering questions (giving the same rating to each of the questionnaire questions throughout or too fast in answering all questions) and were therefore removed from data analysis, resulting in 87 participants.

The participants’ average age was 9.8 (*SD* = 1.26); 55% were boys, and 45% were girls. The participants were racially diverse, with 32.2% of children identifying as Hispanic or Latino, 28.7% as African American, 10.3% as Caucasian, 9.2% as mixed race, 5.7% as Asian, and 13.8% of participants identifying as American Indian/Alaska Native or of Other race. As indicated in Table 1, participants were divided by a median split of a BMI percentile of 75. No differences were identified for age (*p* = 0.919), sex (*p* = 0.831), race (*p* = 0.170), and SD (*p* = 0.458) for children in different BMI percentile groups across the three conditions. The key differences between the ≤75th BMI percentile or >75th BMI percentile, as expected, were participant weight, BMI, and BMI percentile (*p*s < 0.001).

**TABLE 1:** Participant demographics and social desirability results (*N* = 87).

	≤75th BMI percentile (*n* = 44)			>75th BMI percentile (*n* = 43)			*p*
	
	Condition A (overweight/obese) (*n* = 11)	Condition B (average) (*n* = 21)	Condition C (athletic slim) (*n* = 12)	Condition A (overweight/obese) (*n* = 13)	Condition B (average) (*n* = 18)	Condition C (athletic slim) (*n* = 12)	
Age, mean (*SD*)	9.8 (1.3)			9.8 (1.2)			0.919
Weight (kg)	33.8 (6.7)			48.7 (10.3)			<0.001
BMI, kg/m^2^, mean (*SD*)	16.6 (1.5)			23.0 (3.7)			<0.001
BMI percentile, mean (*SD*)	43.5 (22.2)			91.6 (6.3)			<0.001
Sex (boy/girl, n)	8/3	12/9	5/7	9/4	11/7	3/9	0.831
Race (*n*)							0.170
Asian	2	0	0	1	2	0	
African American	1	5	3	5	4	7	
American Indian/Alaska Native	0	1	1	0	0	0	
Caucasian	4	3	0	2	0	0	
Hispanic or Latino	2	7	4	2	10	3	
Mixed	1	2	3	1	1	0	
Other	1	3	1	2	1	2	
Social desirability (*SD*)	3.3 (0.8)			3.4 (0.9)			0.458

A two-way between-subject multivariate analysis of variance indicated a significant Condition effect, *F*(10,154) = 2.418, *p* = 0.011; Wilks’ Λ = 0.747, and a borderline significant interaction effect between BMI percentile and Condition on the combined dependent variables, *F*(10,154) = 1.702, *p* = 0.085; Wilks’ Λ = 0.811. To investigate the impact of each effect on the individual outcome variables, a univariate *F*-test using an alpha level of 0.05 was performed. The general linear model identified an interaction effect (BMI percentile × Condition) for NE (*F* = 4.58; *p* = 0.013; $\eta_{\text{p}}^{2}$ = 0.102), WI (*F* = 5.85; *p* = 0.004; $\eta_{\text{p}}^{2}$ = 0.126), and PA motivation (*F* = 3.17; *p* = 0.047; $\eta_{\text{p}}^{2}$ = 0.073). Pairwise comparisons found that condition A resulted in higher NE and WI among children of >75th BMI percentile than children of ≤75th BMI percentile (*p*s < 0.029). Condition B resulted in higher NE and PA motivation in children of ≤75th BMI percentile than those in >75th BMI percentile (*p* < 0.05). Among children in ≤75th BMI percentile, condition B showed higher NE, WI, AVG play motivation than conditions A and C (*p*s < 0.05). Condition C also resulted in a lower PA motivation than condition B. As for children in >75th BMI percentile, condition A resulted in higher NI, NE, WI, and PA Motivation than conditions B and C, and borderline higher AVG motivation than condition C (*p* = 0.064). See Table 2 for details. Similar results were obtained when controlling for SD and would not be double presented for space conservation.

**TABLE 2:** Means of the main outcome variables per group (*N* = 87).

	Condition A (overweight/obese)	Condition B (average)	Condition C (athletic slim)
	
≤75th BMI percentile	*n* = 11	*n* = 21	*n* = 12
Narrative immersion (NI)	3.39 (0.60)	3.42 (0.59)	3.12 (0.58)
Narrative engagement (NE)	3.19 (0.61)	3.74 (0.50)^#,*a*^	3.10 (0.54)
Wishful identification (WI)	3.09 (1.03)	3.86 (1.20)^a^	3.11 (1.04)
AVG play motivation (AVGM)	3.56 (1.16)	4.35 (0.82)^a^	3.35 (1.36)
PA motivation (PAM)	3.84 (0.69)	4.27 (0.68)^#^	3.57 (0.79)^b^

**>75th BMI percentile**	***n* = 13**	***n* = 18**	***n* = 12**

Narrative immersion (NI)	3.74 (0.47)^c^	3.15 (0.59)	3.13 (0.80)
Narrative engagement (NE)	3.73 (0.48)*^,c^	3.34 (0.68)	3.12 (0.77)
Wishful identification (WI)	4.41 (0.78)*^,c^	3.50 (1.02)	3.33 (1.33)
AVG play motivation (AVGM)	4.15 (0.93)^d^	3.96 (0.96)	3.35 (1.33)
PA motivation	3.93 (0.76)^c^	3.39 (0.90)	3.15 (0.66)

Pairwise comparisons: the different scores are marked with a superscript symbol. *^,#^Different between BMI percentile groups.

*^*a*^Higher than Conditions A and C. ^*b*^Lower than Condition B. ^*c*^Higher than Conditions B and C. ^*d*^Borderline higher than Condition C.*

**FIGURE 2:**
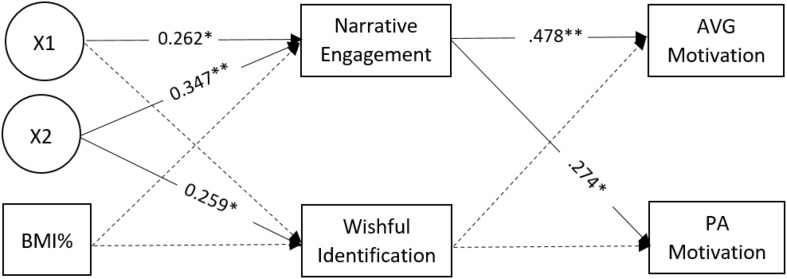
Structural Equation Model for the relation between character body shape, children’s BMI percentile, AVG motivation and PA motivation, with narrative engagement and wishful identification as the mediating variables. X1: the overweight/obese condition in reference to athletic slim condition. X2: the average condition in reference to athletic slim condition. All paths’ estimates are standardized. For the sake of simplicity, the direct and indirect paths from the IVs to the DVs as well as the correlations between the mediators and DVs are not shown. ^∗^*p* < 0.05; ^∗∗^*p* < 0.001.

In terms of the structural equation modeling analysis, Figure 2 presents the original hypothesized model examining the mediating role of NE and WI of the effect of condition of three types of character body shape manipulation (IV) and children’s BMI percentile (IV) on children’s AVG (DV) and PA motivation (DV). For the 3-type character body shape manipulation IVs, two dummy variables (X1 and X2) were created with the “athletic slim” group as a reference group. The model (χ_18_^2^ = 104.37, *p* < 0.01) fits were good (CFI/TLI > 0.96, RMSEA < 0.06, SRMR < 0.08). Both the overweight/obese condition and the average condition (compared to the athletic thin condition) were significantly associated with NE, which was significantly associated with both of the DVs. The indirect effects of the average body image condition (compared to the athletic thin condition) on AVG (*p* = 0.019) and PA motivation (*p* = 0.046) via NE were also significant. Since nearly all of the paths related to WI and the children’s BMI percentile (IV) were non-significant, these two variables were removed from the mediation model, leaving the character body shape as the only IV and the NE as the only mediating variable. Participant age and sex were not significantly associated with the mediators and outcome variables in this model and thus excluded in the final model.

**FIGURE 3:**
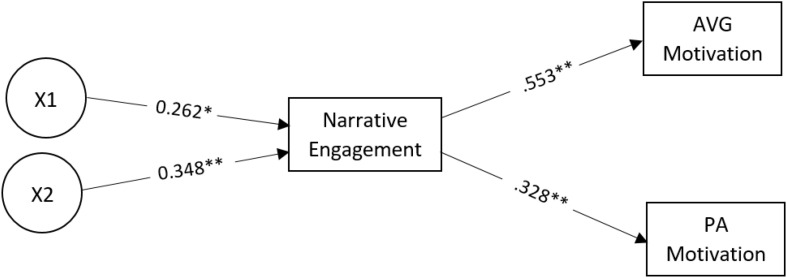
Structural Equation Model for the relation between character body shape and AVG motivation and PA motivation, with narrative engagement as the mediating variable. All paths’ estimates are standardized. X1: the overweight/obese condition in reference to athletic slim condition. X2: the average condition in reference to athletic slim condition. For the sake of simplicity, the direct and indirect paths from the IVs to the DVs as well as the correlations between the DVs are not shown. ^∗^*p* < 0.05; ^∗∗^*p* < 0.001.

Figure 3 has the updated mediation structural equation model. The updated model (χ_9_^2^ = 67.48, *p* < 0.01) has excellent goodness of fit indices (CFI/TLI > 0.98, RMSEA < 0.04, and SRMR < 0.06) and explains approximately 37 and 18% of the variances of children’s AVG and PA motivation, respectively. In this model, the overweight/obese (β = 0.262, *p* = 0.03) and average (β = 0.348, *p* = 0.002) body image conditions were significantly associated with NE. The direct effects of NE on children’s AVG motivation (β = 0.553, *p* < 0.001) and PA motivation (β = 0.328, *p* < 0.01) remained significant. While the indirect effects of the overweight/obese body image condition (compared to the athletic thin condition) on AVG (*p* = 0.058) and PA motivation (*p* = 0.085) via NE were marginally significant, the indirect effects of the average body image condition (compared to the athletic thin condition) on AVG (*p* = 0.019) and PA motivation (*p* = 0.046) via NE remained significant, indicating the robustness of this particular effect.

To check the robustness of the overall model, two-way interactions of SD with the condition variables were tested. These interaction coefficients were not significant and results overall remained similar.

## Discussion

To increase children’s engagement in PA-inducing AVGs, we investigated the effects of manipulating the character body shape in an animated narrative on children’s NI, NE, WI, AVG, and PA motivation.

We have found that the visual presentation of different character body sizes in a narrative impacted children’s NE across different weight groups. Children with a higher than 75th BMI percentile had a significantly greater NI, NE, WI, and PA motivation in the overweight/obese (condition A) character condition than those who were in the average or athletic slim conditions (conditions B and C). On the other hand, children equal to or below the 75th BMI percentile had higher NE, WI, and AVG play motivation when the characters body shape was average (condition B) than in condition A (overweight/obese) or C (athletic slim). Furthermore, the overweight/obese character body shape (condition A) resulted in higher NE and WI among children with higher than 75th BMI percentile than those with 75th or less BMI percentile while condition while the average character body shape (condition B) resulted in higher NE and PA motivation in the 75th or less BMI percentile than those whose BMI percentile are 75th or higher. Last but not least, the NE had a stable and significant mediating effect between the character body manipulation and the AVG play motivation and physical activity motivation.

In the mediation model, the NE repeatedly demonstrates the capability of narratives to translate the power of entertainment to health behavior change. We have consistently demonstrated the importance of the engaging factor, that is, a health intervention, be it in the form of narrative media or not, must be engaging to have the potential to induce health behavior change. On the other hand, this also suggested the psychological crux of successful narrative health intervention for children. A narrative message must be fully understood, grabbing children’s attention, capturing their emotion, and drawing them into the narrative world.

Wishful identification yielded no significant mediating path results. While this could be potentially attributed to the relatively small sample of the study, the result also suggests that children’s engagement with a story may be more relevant to their motivation to exercise than their desire to become like the characters presented in the story. In addition, the original WI scale adopted in this study did not include items asking about the physical characteristics of the characters, incorporation of these items may alter the results.

While WI was not found to have significant effects on children’s PA or AVG play motivation in this study, this concept can still be relevant to a child’s engagement with the story and thus the relationship between WI and NE should be further explored. For example, children showed significantly higher WI with characters that looked like their own body shapes in both weight groups. Since these characters may already appear more similar to the children, the increased desire to be like the shape of those characters may not translate directly to exercise behaviors among the audience, despite the fact these characters themselves are engaged in physical activities in the animated story. On the other hand, when WI was higher, so was NE, which according to the mediation results did mediate AVG and PA motivation. A higher WI may contribute to higher NE as well as the health behavior intentions in an indirect fashion.

From a practical perspective, given the higher NE, NI, and WI presented when characters have body shapes that match the intended audience, when executing potential PA interventions among children population, media creators should be conscious of creating body shapes of various sizes if they are catering to diverse children body shapes. Creating the appropriate visual aspect of the character body shape could not only increase children’s engagement with the story, but also motivate them to participate in the physical activities suggested and potentially enhance their adherence to the intervention. This way, children of all body shapes can have access to an engaging and relatable narrative.

Due to the importance of NE in PA intervention, and potential clinical use, further research is needed on how to best engage children of various backgrounds to ensure the narrative truly serves the entire child population. For example, while we have found that the children in our population were more likely to be engaged in stories with similarly sized characters, in the study design; however, we did not account for gender differences as our presented characters had one male and one female character, both of whom were presented in a similar fashion in each of the body shape conditions. As previously reviewed, the traditional gender representation in media is more likely to have women being represented as underweight and men presented as muscular (Barlett et al., 2008). Thus, the way the body shape differences are represented across different gendered characters still needs to be investigated in terms of their differing effects on NE and exercise motivation.

In addition, we have only presented the character’s body shape in an initial segment of a long story, most of the effects observed here are based on the beginning status of the character body shape. Will there be a differential response when the characters’ body shapes start to increase or decrease in accordance with children’s exercise participation over different time intervals (Fox and Bailenson, 2009)? The dynamics of the characters’ body shape change mirroring or opposite to the audience’s own body shape during the narrative development deserve some additional exploration.

It is also interesting to observe that the athletic slim body type of cartoon characters did not appeal to children in either weight group with the 75th BMI percentile as the cutoff point. While dividing children’s weight groups in a much more fine-tuned fashion may allow us to identify some children with less BMI percentile to engage with the athletic slim condition more, further examination of the data among the slim children proved otherwise; we could not identify a significant number of children who gave this condition higher ratings. This may have something to do with potentially visual and aesthetic fatigue with respect to children’s long-term exposure to the stereotyped media characters’ body shape presentation (Lemish and Johnson, 2019). Therefore, when they are presented with someone without the stereotyped body shape, the novelty of such visual presentation may be more welcome among them. An alternative explanation is that the increasing obesity epidemic, coupled with popular culture’s influence on body ideals change over the past several decades, may also have already shifted the social and perceptual norms of a typical child. Though research in this area has been sparse.

This research project is not without limitations. The participants only watched 15 min of the narrative and did not watch the full six-season animated series over time. Thus, it is unclear if the results would be altered should the children have been exposed to more episodes and more viewing sessions. In addition, due to space and time constraints, we have only measured participants’ response through survey questionnaires. Children did not actually play the AVG after watching the episodes. Thus, exercise motivation was determined based on self-report instead of objectively measured PA outcomes. More research is needed regarding the actual increase in AVG playtime instead of hypothetical change reported on paper. Our access to children was also constrained by the schedule of different afterschool activities in different classes, resulting in relatively limited sample size, which could contribute the borderline and lack of significant results. Similarly, although our participant base was racially diverse and relatively equally divided between boys and girls, the small sample size may preclude us from in-depth examination of specific racial and sex characteristics and differences.

Despite these limitations, this project found that narrative cartoon characters that mirror the target participant’s body shape can increase NI, WI, and NE. NE in turn mediates the effect of character body shape manipulation on AVG and PA motivation. Such findings strongly suggests the importance of having relatable characters to increase children engagement with narrative health interventions. Future health narrative content producers should identify optimal strategies in character body shape design to encourage children of different weight status to participate in PA with engaging stories to maximize health narratives’ persuasive potentials.

## Data Availability Statement

The raw data supporting the conclusions of this article will be made available by the authors, without undue reservation.

## Ethics Statement

The studies involving human participants were reviewed and approved by Northeastern University IRB. Written informed consent to participate in this study was provided by the participants’ legal guardian/next of kin.

## Author Contributions

DA contributed to data analysis and wrote the first draft of the manuscript. CS participated in data analysis and results interpretation. AL designed the study, supervised the data collection, participated in data analysis, results interpretation, and supervised the study. All the authors contributed to the manuscript writing, read and approved the final version of the manuscript.

## Conflict of Interest

The authors declare that the research was conducted in the absence of any commercial or financial relationships that could be construed as a potential conflict of interest.

## Supplementary Material

Supplementary Table 1 Shapiro–Wilk and Levene’s tests results.
