# Supplementary material for: What Type of Body Shape Moves Children? An Experimental Exploration of the Impact of Narrative Cartoon Character Body Shape on Children’s Narrative Engagement, Wishful Identification, and Exercise Motivation
Source: Front Psychol. 2021 Jul 12;12:653626. doi: 10.3389/fpsyg.2021.653626 (PMC8312721; doi:10.3389/fpsyg.2021.653626)
Supplement: Supplementary Table 1 — Shapiro–Wilk and Levene’s tests results. [file Table_1.pdf]

Table S1

Shapiro-Wilk and Levene’s tests results.

Variable	Shapiro-Wilk	Levene
Age	0.938	0.558
Weight	0.962	0.902
BMI	0.912	0.751
BMI percentile	0.886	0.683
SD	0.964	0.804
Narrative Immersion	0.973	0.932
Narrative Engagement	0.974	0.904
Wishful Identification	0.918	0.816
AVG Play Motivation	0.900	0.554
Physical Activity Motivation	0.952	0.489
